# Methylome and transcriptome analyses reveal HLA-DMB’s contribution to periodontitis development

**DOI:** 10.1371/journal.pone.0319055

**Published:** 2025-04-23

**Authors:** Bo Zhao, Ronghua Li

**Affiliations:** Department of Stomatology, Tianjin First Central Hospital, Tianjin, P.R. China; High Point University, UNITED STATES OF AMERICA

## Abstract

**Background:**

Periodontitis is a typical oral disease. Polymorphonuclear neutrophils (PMNs) are crucial immune cells in periodontal tissues, relating to infection, inflammation, and innate immunity. We herein aimed to explore important periodontitis PMN related genes.

**Methods:**

Periodontitis and control samples were downloaded from Gene Expression Omnibus database, including GSE173082 (methylation data, n=72), GSE10334 (n=127), GSE43525 (n=23), GSE16134 (n=134). Differential expression analysis and differential methylation analysis was employed to find candidate genes. Receiver operating characteristic analysis was performed to evaluate the diagnostic value of the hub gene. The functional pathways were determined by gene set enrichment analysis. Using CIBERSORT software, the immune cell infiltration landscape of periodontitis tissue was explored. The mRNA and protein levels of target gene in clinical tissue samples were determined employing RT-qPCR and western blotting. All statistical analyses were conducted in R software.

**Results:**

After integrating DNA methylation with transcriptome profiles, GRASP, HLA-DMB, HLA-DMA, CAB39, NCOA2 and TLE4 were identified as candidate genes in periodontitis PMNs. HLA-DMB showed the highest correlation with core DNA methyltransferase DNMT3B (p < 0.05). Between high and low HLA-DMB expression samples, multiple immune related pathways were enriched, and differential immune cell infiltration was observed (p < 0.05). HLA-DMB exhibited significantly higher expressions in both public database and clinical tissue samples (p < 0.05). HLA-DMB was a diagnostic marker for periodontitis (GSE43525 AUC=0.777 and GSE16134 AUC=0.783).

**Conclusions:**

Significantly higher HLA-DMB expression was noticed in PMNs of periodontitis, which probably contributed to the development of periodontitis. HLA-DMB is a promising diagnostic marker for periodontitis.

## 1. Introduction

Periodontitis, as a local chronic inflammatory condition, usually manifests as gingival bleeding and supporting tissue destruction, with a high global prevalence worldwide [1]. Edentulism may arise from periodontitis’ destruction of the alveolar bone or periodontal supporting tissues [2,3]. Recently, although many studies have focused on the periodontal microbiota and host factors, deepening pathogenic mechanisms of periodontitis are still largely unknown.

Polymorphonuclear neutrophils (PMNs) are crucial immune cells in periodontal tissues, exerting important roles in the infection, inflammation, and innate immunity of periodontitis [4]. Neutrophils are the first group of immune cells recruited to the infected site, which are traditionally considered as professional phagocytic and acute inflammatory cells engulfing microbial pathogens [5,6]. PMNs migrate to the infected sites quickly and eliminate periodontal pathogens at the beginning stage, mainly through the following four functions, including phagocytosis, release of reactive oxygen species (ROS), release of neutrophil extracellular traps (NETs) and release of neutrophil granules [7]. Moonen et al. have demonstrated the a positive correlation between oral PMNs and oral inflammation severity [8]. However, earlier study has indicated that the overactivation of PMNs during the immunological response to periodontitis might result in tissue damage [9]. A recent case-control work has implied that there are indeed higher numbers of oral PMNs and degranulation markers in periodontitis [10]. Moreover, the role of PMNs are found to transfer as neutrophil-derived antigen-presenting cells (nAPC) via the expression of classical dendritic cells (cDC) surface markers, such as MHCII, in patients with infections and autoimmune disorders [11]. More recent study has revealed PMN’s role in producing various cytokines/chemokines/growth factors for immune modulation, in inflammatory diseases and cardiovascular diseases [6,12]. Whereas, to the best of our knowledge, the potential underlying mechanisms relating PMNs to the onset/ progression of periodontitis still remain to be clarified.

Periodontitis has been known to involve in an interaction between bacteria, inflammation, host response genes, and environmental factors [13]. Of which, epigenetic changes like special DNA methylation patterns are found to dampen or promote the inflammatory response to bacterial challenge in periodontitis patients [13]. Under this context, several studies have indicated the potential associations between DNA methylation and periodontitis development as well as systemic diseases [14]. Promoter regions of pro-inflammatory genes like interleukin (IL)-6, IL-8, INF-γ have been found in hypomethylated status in human gingival biopsies [15]. Moreover, Barros et al. have documented that in periodontitis patients, there are differentially methylated CpG sites in thioredoxin gene, which is relating to innate immune response activation [16]. On the other hand, DNA methylation has recently been indicated to affect the function of PMNs through modulating gene expression. For instance, in neutrophils of lupus patients, there is significant abnormal demethylation of interferon signature genes, probably involving the pathogenesis of lupus [17]. Nevertheless, as far as we know, rare studies have focused on the DNA methylation pattern in PMNs of periodontitis.

Hence, integrating transcriptome data and DNA methylation data of periodontitis, the purpose of this work is to explore the potential hub genes with methylation modification as well as the special DNA methylation pattern in PMNs of periodontitis. We hypothesized that the hub gene with distinct DNA methylation could contribute to the progression of periodontitis. Our findings are promising to give deeper insights into the pathogenic mechanisms of the onset of periodontitis, meanwhile to provide more reference information for the early detection of periodontitis.

## 2. Materials and methods

### 2.1. Data resources

Four periodontitis related datasets were obtained from the Gene Expression Omnibus (GEO, https://www.ncbi.nlm.nih.gov/geo/) database. GSE173082 methylation dataset comprised 36 healthy samples and 36 periodontitis samples (Infinium MethylationEPIC). GSE10334 contained 64 healthy samples and 63 periodontitis samples (Affymetrix Human Genome U133 Plus 2.0 Array). GSE43525 dataset included 23 PMN samples, of which 7 healthy samples and 16 periodontitis samples (Illumina HumanHT-12 V4.0 expression beadchip). GSE16134 contained 69 healthy samples and 65 periodontitis samples. The detailed information of samples in above datasets have been summarized in S1 Table.

### 2.2. Differential expression analysis

The differential expression analysis was conducted between periodontitis vs. control, based on GSE43525 and GSE10334 in the limma package (version 3.56.2, [18]) of R language (version 4.2.0, the same below) (statistically screening criteria: |Log_2_FC| >0.5 and P value <0.05). In this function package, the expression matrix and grouping information were used as input files, to obtain differentially expressed gene list.

The methylation profiling dataset GSE173082 was analyzed by using the ChAMP package [19] of R language to identify the differentially methylated CpG sites (DMSs) (statistically screening criteria: P value <0.05). Herein, the input files were grouping information and the β value of methylated cg sites. The DMS were mapped to the corresponding genes and genomic regions to confirm the differentially methylated genes (DMGs).

### 2.3. Correlation analysis

The correlations between candidate genes and DNA methyltransferase were performed using Pearson’s correlation analysis.

### 2.4. Clinical sample collection

A total of 15 cases of periodontitis tissues and 15 cases of healthy controls were collected from September 20 to November 10, 2024 in Tianjin First Central Hospital (Tianjin, China).

Our study was approved by the Ethics Committee of Tianjin First Central Hospital, in accordance with the Declarations of Helsinki. All participants signed for informed consent voluntarily.

### 2.5. Reverse transcription quantitative polymerase chain reaction (RT-qPCR) assay

TriQuick Reagent Total RNA extraction reagent (R1100, Solarbio, Beijing, China) was utilized to extract total RNA. After reverse transcription, PCR amplification was conducted using 2×SuperStar Universal SYBR Master Mix (CW3360, CWBIO, Jiangsu, China), under the following protocol: pre-denaturation at 95°C for 30s, 40 cycles of 95°C for 10s and 60°C for 30s. The GAPDH was used as the internal reference, and the primers have been summarized in Table 1. The relative mRNA level was calculated according to 2^−ΔΔCt^ [20].

**Table 1:** Primer sequences for qPCR.

Genes	Forward Primer (5’-3’)	Reverse Primer (5’-3’)
HLA-DMB	TGGGGTGCTGAATAGCTTGG	GGGTGTGTGTGGCACAATTC
GAPDH	GAAGGTGAAGGTCGGAGTC	GAAGATGGTGATGGGATTTC

### 2.6. Western blot assay

The total protein in tissue samples were extracted using RIPA buffer (R0010-100ml, Solarbio) and then quantified by the BCA kit (CW0014S, CWBIO). The western blot was then conducted in previously reported methods [21]. The antibodies used in our study were listed as below, including anti-GAPDH antibody (1:10000, No. 60004–1-Ig, Proteintech, Chicago, IL, USA), anti-HLA-DMB antibody (1:1000, No. 21704–1-AP, Proteintech), horseradish peroxidase-labeled goat anti-rabbit IgG (H+L) (1:10000, ZB-2301-0.1ml, ZSGB-BIO, Beijing, China), and horseradish peroxidase-labeled goat anti-mouse IgG (H+L) (1:10000, ZB-2305-0.1ml, ZSGB-BIO, Beijing, China). The gray values were analyzed in ImageJ software.

### 2.7. Immune cell infiltration analysis

CIBERSORT software [22] was used to calculate the composition and relative proportions of infiltrating immune cells in each sample from various groups. In this software, deconvolution algorithm was employed to characterize the composition of infiltrating immune cells according to the 547 preset barcode genes. The proportions of all estimated immune cell types in each sample summed to 1.

### 2.8. Functional enrichment analyses

Regarding candidate genes’ functional information, it was conducted using clusterProfiler of R. Moreover, the Gene Set Enrichment Analysis (GSEA) was employed to obtain the functional information between different periodontitis groups, using the ReactomePA package and clusterProfiler package (version 4.8.3 [23]) of R based on the gene set msigdb.v7.5.1.entrez.gmt. Pathways with p<0.05 were taken as significantly enriched.

### 2.9. Protein protein interaction (PPI) network analysis

STRING database (https://cn.string-db.org/) is a powerful tool including wide protein-protein interactions, involving both physical interactions as well as functional associations [24]. The PPI network was built to predict the interactions employing the STRING tool (version 11.0 [25]), which was then visualized in cytosacpe software (version 3.7.2 [26]).

### 2.10. Clinical value evaluation of hub gene in periodontitis

To evaluate the clinical value of the target gene, receiver operating characteristic (ROC) analysis and the calculation of the area under the curve (AUC) were performed using the pROC package of R language. Besides, the potential drugs targeting the hub gene were also predicted in oncoPredict of R, and the Pearson’s correlation analysis between potential drugs and the hub gene was done.

## 3. Results

### 3.1. Identification of candidate genes in PMNs of periodontitis

**Fig 1:**
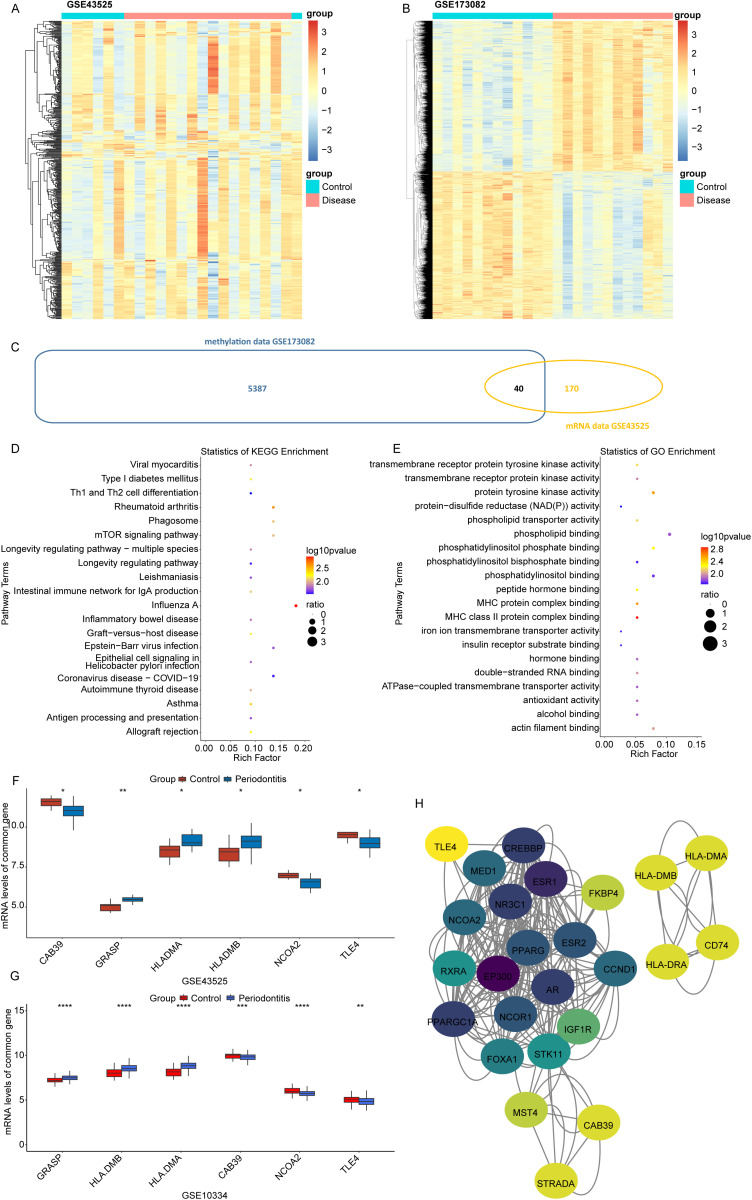
Identification of candidate genes correlated with PMNs in periodontitis. (A) DEGs identified in GSE43525 dataset. (B) DMGs identified in GSE173082 dataset. (C) Venn diagram of 210 DEGs and 5427 DMGs. (D-E) The top 20 significant KEGG pathways and GO terms based on 40 overlapping genes, respectively. (F-G) The expression of 6 candidate genes in GSE43525 and GSE10334 datasets, respectively. (H) The interaction network among the candidate genes and their related genes.

To identify the crucial genes in PMNs of periodontitis, we first performed the differential expression analysis based on GSE43525 dataset. Totally 210 differentially expressed genes (DEGs) were identified in periodontitis samples compared to control samples, including 58 up-regulated genes and 152 down-regulated genes (Fig 1A, S2 Table). In methylation profiling GSE173082, compared to control samples, we found 13860 differential CpG sites and confirmed 5427 corresponding DMGs in periodontitis samples, among which 2853 genes were hypermethylated, and 2574 were hypomethylated genes (Fig 1B). Then 210 DEGs and 5427 DMGs were cross-analyzed, and 40 overlapped genes were obtained (Fig 1C). The functional enrichment analysis was then conducted on these 40 genes, indicating that these genes were significantly enriched in 27 KEGG pathways and 54 GO terms (p <0.05, S3 Table). The top 20 terms were displayed in Fig 1D, 1E, separately.

In an independent cohort GSE10334, we found that 6 candidate genes among the above 40 overlapped genes showed both significant expression differences and the same expression tendency in periodontitis samples, consistent with the result in GSE43525 (Fig 1F), including GRASP, HLA-DMB, HLA-DMA, CAB39, NCOA2 and TLE4 (Fig 1G). The interaction analysis was performed on the 6 genes and their related genes. We found that direct interaction was observed between HLA-DMB and HLA-DMA, while indirect interactions among CAB39, NCOA2 and TLE4 were observed (Fig 1H).

### 3.2. Periodontitis related hub gene HLA-DMB was mainly regulated by methyltransferase DNMT3B

**Fig 2:**
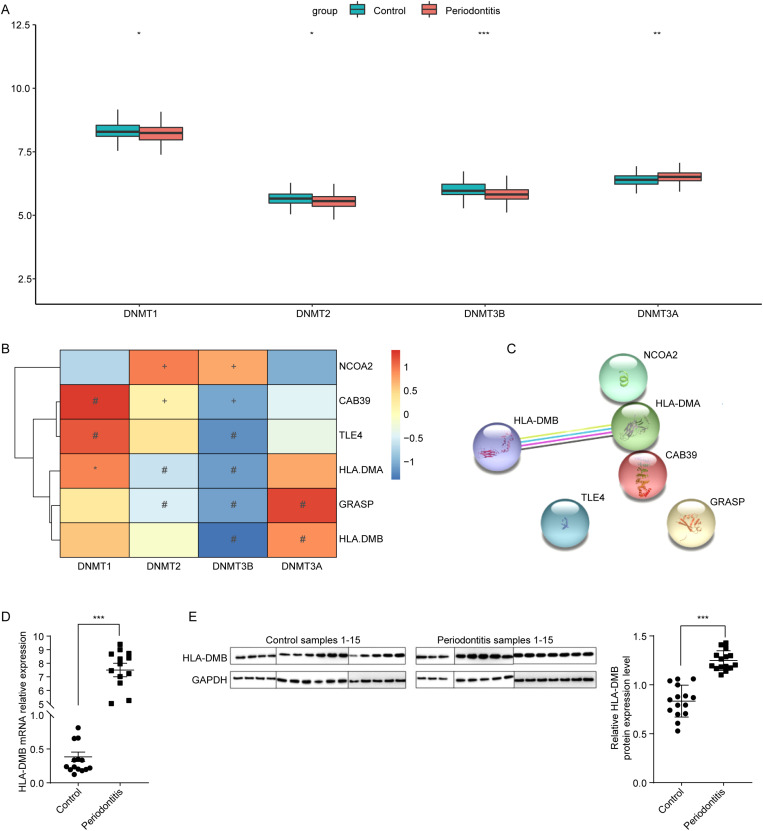
The results of correlation analysis between candidate genes and methyltransferases. (A) Differentially expressed methyltransferases between periodontitis and normal samples. (B) Correlation between differential methyltransferases and candidate genes. (C) PPI network of 6 candidate genes. (D-E) The mRNA and protein levels of HLA-DMB were determined employing RT-qPCR and western blotting, separately. *** p <0.001.

To further explore the hub genes among the above differentially expressed as well as differentially methylated candidate genes, the methyltransferases’ expressions were analyzed based on periodontitis related data in GSE10334. Compared to control samples, the methyltransferases DNMT1, DNMT2 and DNMT3B were significantly downregulated in periodontitis samples, while significantly higher DNMT3A expression was observed in periodontitis samples (Fig 2A).

Thereafter, the correlation between methyltransferases DNMT1, DNMT2, DNMT3B, DNMT3A and the 6 candidate genes were analyzed. Our results showed that DNMT3B was significantly negatively correlated with GRASP, HLA-DMB, HLA-DMA, CAB39, and TLE4 (Fig 2B), implying the predominant role of DNMT3B in periodontitis. Among all 6 candidate genes, HLA-DMB showed the strongest correlation with DNMT3B, which was selected for further analyses. The protein-protein interaction (PPI) network analysis of 6 candidate genes suggested that multiple interactions between HLA-DMB and HLA-DMA could be observed (Fig 2C).

Next, to further validate the expression of HLA-DMB, we have collected the locally clinical samples. The mRNA and protein levels of HLA-DMB in clinical tissue samples were determined employing RT-qPCR and western blotting. The results indicated that compared to control samples, the mRNA and protein levels of HLA-DMB were both significantly elevated in periodontitis specimens (p <0.001, Fig 2D, 2E), in line with the data basing on public database.

### 3.3. Immune cell infiltration landscape of periodontitis

**Fig 3:**
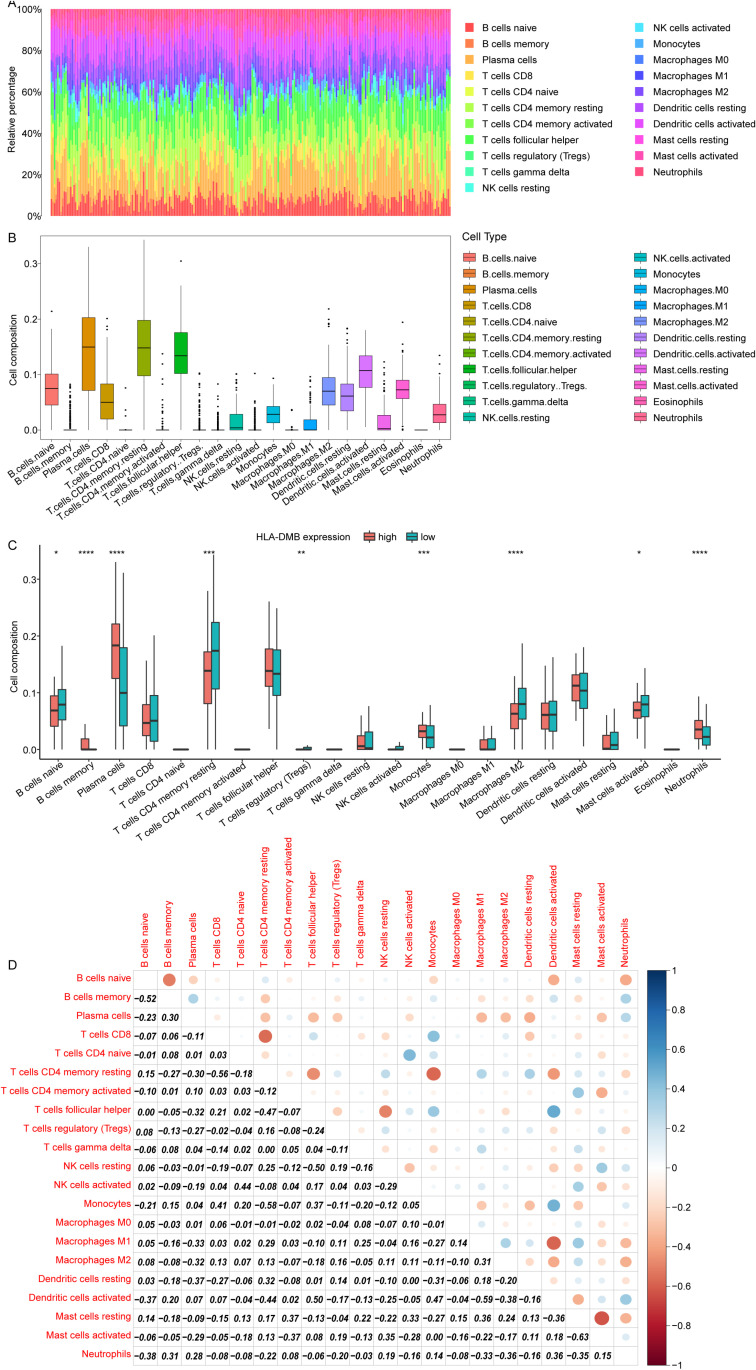
Immune cell infiltration landscape of periodontitis. (A) The relative infiltration proportions of immune cells. (B) The composition of infiltrating immune cells. (C) Relative proportions of 22 kinds of immune cells in the high and low HLA-DMB expression periodontitis samples. (D) The correlation analysis of 22 kinds of immune cells.

Subsequently, HLA-DMB related immune landscape in periodontitis was analyzed based on GSE10334 cohort. All periodontitis samples were divided into the high and the low expression group, according to the median HLA-DMB expression. The relative infiltration proportions and composition of infiltrating immune cells showed heterogeneity among various periodontitis samples (Fig 3A). The plasma cells, CD4 memory resting T cells, and follicular helper T cells exhibited the highest relative infiltration proportions in periodontitis samples (Fig 3B). Compared to the low HLA-DMB expression periodontitis samples, significantly higher proportions of B cells memory, plasma cells, monocytes and neutrophils were observed, but lower proportions of B cells naïve, T cells CD4 memory resting, Macrophages M2 and Mast cells activated were found in high HLA-DMB expression group (Fig 3C). Then, correlation analysis all immune cells in periodontitis suggested that neutrophils were positively correlated with B cells memory and dendritic cells activated (Fig 3D). Neutrophils showed negative correlation with B cells naïve, macrophages M1, macrophages M2 and mast cells resting (Fig 3D).

### 3.4. Functional pathways affected by HLA-DMB in periodontitis

**Fig 4:**
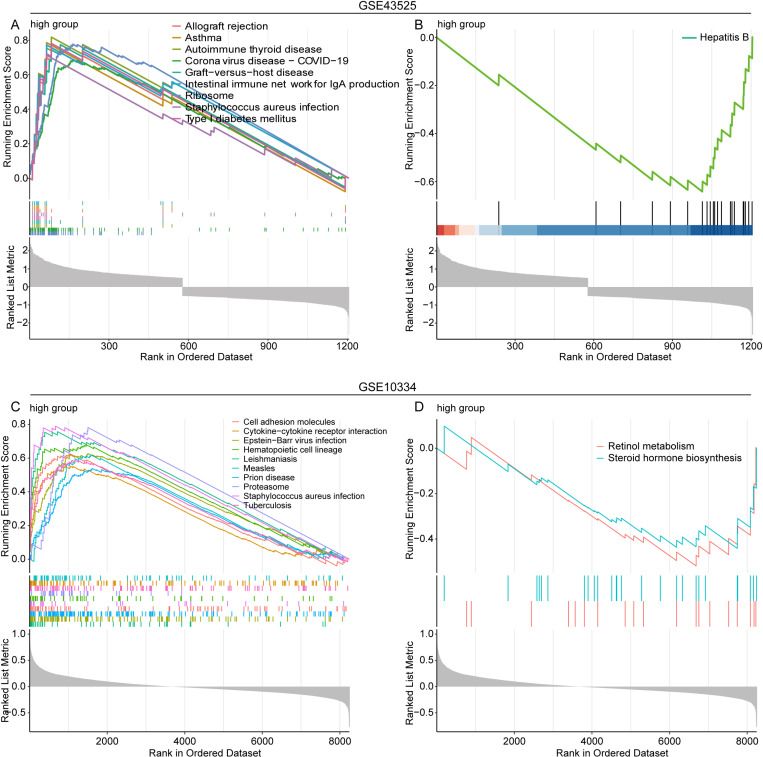
The significantly enriched pathways in the high group compared with the low group. (A) Nine significantly activated pathways (GSE43525). (B) One significantly inhibited pathway (GSE43525). (C-D) Top 10 significantly activated pathways and 2 significantly inhibited pathways (GSE10334). ES, enrichment score; NES, normalized ES; NOM p-val, normalized p-value.

In GSE43525 dataset, all samples were ranked in HLA-DMB expression descending order, then the top 25% and bottom 25% samples were considered high and low HLA-DMB expression groups, respectively. The GSEA results indicated that the 65 pathways were significantly enriched in the high HLA-DMB expression group compared to the low HLA-DMB expression group (P value < 0.05, S4 Table). The top 10 most significant pathways were displayed, including 9 activated pathways (Fig 4A) and 1 inhibited pathway (Fig 4B). Similar analysis was also conducted in GSE10334, we found that totally 131 signaling pathways were significantly activated (Fig 4C, top 10 pathways, S5 Table), while 2 pathways were obviously inhibited (Fig 4D) in high HLA-DMB expression group.

### 3.5. The potential clinical value of HLA-DMB in periodontitis

**Fig 5:**
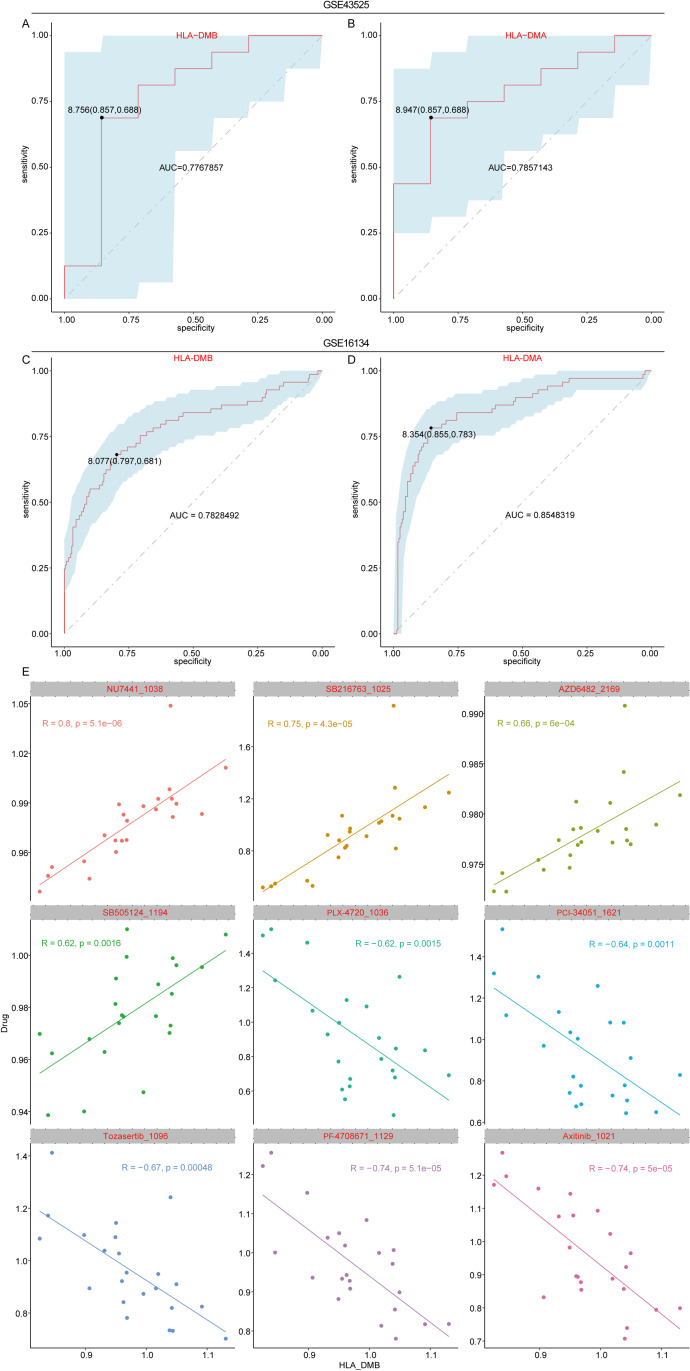
The results of ROC analysis and drug prediction. (A-B) ROC curves of HLA-DMB and HLA-DMA, basing on GSE43525. (C-D) ROC curves of HLA-DMB and HLA-DMA, basing on GSE16134. (E) The correlation between HLA-DMB and potential drugs.

To further confirm the diagnostic value of HLA-DMB for periodontitis patients, we performed ROC analysis in both GSE43525 and GSE16134 datasets. We found that HLA-DMB was a potential diagnostic marker in periodontitis in both datasets (Fig 5A, AUC=0.777; Fig 5C, AUC=0.783). Considering the interactions between HLA-DMB and HLA-DMA in periodontitis, the ROC analysis was also conducted on HLA-DMA, which indicated that HLA-DMA displayed a good performance on distinguishing periodontitis from normal samples (Fig 5B, AUC=0.786; Fig 5D, AUC= 0.855).

Additionally, we have predicted the potential drugs targeting HLA-DMB. We found that HLA-DMB was significantly positively correlated with NU7441_1038, SB216763_1025, AZD6482_2169, and SB505124_1194, while significantly negatively correlated with PLX-4720_1036, PCI-34051_1621, Tozasertib_1096, PF-4708671_1129, and Axitinib_1021 (Fig 5C).

## 4. Discussion

During the past years, accumulating evidence has indicated that periodontitis is not only a major oral disease, but is also closely associated with systemic diseases, such as diabetes and atherosclerosis [27]. Increasing systemic inflammatory and immune burden caused by periodontal infection are associated with high risk of systemic disease. [28,29]. Significantly higher HLA-DMB expression and its close correlation with methyltransferase DNMT3B were observed in periodontitis samples, and HLA-DMB was a promising diagnostic marker for periodontitis patients.

PMNs exerted an important role in both health and periodontitis oral circumstance, owing to their essential ability to maintain bacterial community symbiosis and bactericidal effects [9,30]. Moreover, studies on DNA methylation in neutrophils have indicated a profound variation among different individuals [31]. Hence, first, we have integrated periodontitis related DNA methylation data with PMN mRNA data in order to obtain the differentially expressed and methylated genes in periodontitis PMNs. After joint analysis, we noticed that the significant association between DEG HLA-DMB and differential methyltransferase DNMT3B in periodontitis.

DNMT3B, together with DNMT1, DNMT3A is in charge of adding methyl groups to CpG dinucleotides in mammals [32]. DNMT3A and DNMT3B played roles as *de novo* enzymes [33]. Abnormal epigenetic alterations of DNA methylation have been widely investigated in multiple diseases, such as atherosclerosis [34] and cancers [35]. Whereas, aberrant DNMT3B has been rarely directly connected with periodontitis as far as we know. We have first revealed the correlation of DNMT3B and periodontitis PMN related genes. Additionally, recent work has indicated that DNMT3B (rs2424913) polymorphism at the promoter region contributed to a significantly higher susceptibility to periodontitis [36]. Dias et al. have demonstrated the association between the same DNMT3B (rs2424913) polymorphism and periodontitis as well as systemic lupus erythematosus alone or combined with periodontitis [37]. Thus, aberrant DNMT3B gene expression or polymorphism would exert direct or indirect roles in periodontitis. Briefly, the deepening details of DNMT3B can not be concluded in this work, whilst it deserved more exploration in future studies.

Next, we found that the higher expression of HLA-DMB in periodontitis PMN was probably affected by the methylation modification of DNMT3B, due to the significant correlation between them. HLA-DMB belongs to the HLA class II gene, which participated in the moulding of MHC class II immunopeptidomes [38,39]. The potential impacts of HLA-DM proteins’ efficiency in the HLA class II peptide selection have been indicated in several autoimmune diseases’ development, such as diabetes and rheumatoid arthritis [40,41]. However, whether HLA-DMB could exert similar roles in periodontitis as in other autoimmune diseases remains unclear. Moreover, considering the key contribution of HLA-DMB in periodontitis PMN, we found that neutrophils and other 8 types of immune cells were significantly differentially infiltrated between high and low expression periodontitis samples. Neutrophils showed a higher infiltration proportion in highly HLA-DMB expressed periodontitis samples. On the other hand, there were two subtypes of oral neutrophils, including parainflammatory neutrophils and proinflammatory neutrophils [42]. Collectively, we reasonably proposed the hypothesis that HLA-DMB exhibited significantly higher expression under the downregulated modification of methyltransferase DNMT3B, which activated more proinflammatory neutrophils in oral circumstance, thereby contributing to the pathogenesis of periodontitis. Regarding the possible functional way of proinflammatory neutrophils, the enhanced phagocytosis, ROS production, and NET formation might individually or jointly participate in the progression of periodontitis [9]. Accordingly, the role of HLA-DMB should be related to neutrophils/ PMN in periodontitis, although the exact mechanism between them was not clear at present. Meanwhile, combining ROC results and clinical validation of HLA-DMB, it showed great potential in distinguishing periodontitis samples from normal samples, thereby HLA-DMB was a novel detection target in diagnosing periodontitis. The diagnostic value and potential target drugs of HLA-DMB reminded us that more details of HLA-DMB should be paid more attention in our future study.

Although the crucial potential of HLA-DMB in periodontitis has been uncovered, there are still several limitations in this present work. Firstly, public data and clinical specimens have been included in our study, while we have to recognize the possible biases in sample selection, which should be further improved in the subsequent study. Then, as the predominant results were obtained basing on public data, our analysis was partly limited by the available data. Despite preliminary exploration of the functional roles of HLA-DMB, more detailed mechanisms underlying HLA-DMB has not been fully investigated in our present work. In our future study, it is imperative to further decipher the functional pathways of HLA-DMB, thereby to develop novel targeted prevention and treatment strategy for periodontitis patients.

## 5. Conclusions

In conclusion, we have revealed the diagnostic value of HLA-DMB in periodontitis for the first time, combining DNA methylation with transcriptome data. Meanwhile, the role of HLA-DMB in periodontitis is probably modified by the downregulated methyltransferase DNMT3B, leading to attenuated expression-inhibition and higher HLA-DMBexpression, which jointly contributed to the progression of periodontitis. Our findings are conducive to giving deepen insights into the epigenetic modification and clinical application of HLA-DMB in periodontitis.

## Supporting information

S1 Table Detailed clinical information of samples in four GEO datasets. (XLSX)

S2 Table Detailed list of 210 DEGs based on GSE43525 dataset. (XLSX)

S3 Table Full results of functional enrichment analysis based on 40 overlapping genes. (XLSX)

S4 Table All significantly enriched pathways obtained from GSEA based on GSE43525. (XLSX)

S5 Table All significantly enriched pathways obtained from GSEA based on GSE10334. (XLSX)
